# A Study on Congruency Effects and Numerical Distance in Fraction Comparison by Expert Undergraduate Students

**DOI:** 10.3389/fpsyg.2020.01190

**Published:** 2020-06-18

**Authors:** Nicolás Morales, Pablo Dartnell, David Maximiliano Gómez

**Affiliations:** ^1^Department of Psychology, Faculty of Social Sciences, Universidad de Chile, Santiago, Chile; ^2^Department of Mathematical Engineering, Faculty of Physical and Mathematical Sciences, Universidad de Chile, Santiago, Chile; ^3^Center for Advanced Research in Education (CIAE), Universidad de Chile, Santiago, Chile; ^4^Center for Mathematical Modeling (CMM), Faculty of Physical and Mathematical Sciences, Universidad de Chile, Santiago, Chile; ^5^Institute of Educational Sciences (ICEd), Universidad de O’Higgins, Rancagua, Chile

**Keywords:** fractions, fraction comparison, math experts, congruency, gap thinking, numerical distance, numerical cognition

## Abstract

School mathematics comprises a diversity of concepts whose cognitive complexity is still poorly understood, a chief example being fractions. These are typically taught in middle school, but many students fail to master them, and misconceptions frequently persist into adulthood. In this study, we investigate fraction comparison, a task that taps into both conceptual and procedural knowledge of fractions, by looking at performance of highly mathematically skilled young adults. Fifty-seven Chilean engineering undergraduate students answered a computerized fraction comparison task, while their answers and response times were recorded. Task items were selected according to a number of mathematically and/or cognitively relevant characteristics: (a) whether the fractions to be compared shared a common component, (b) the numerical distance between fractions, and (c) the applicability of two strategies to answer successfully: a congruency strategy (a fraction is larger if it has larger natural number components than another) and gap thinking (a fraction is larger if it is missing fewer pieces than another to complete the whole). In line with previous research, our data indicated that the congruency strategy is inadequate to describe participants’ performance, as congruent items turned out to be more difficult than incongruent ones when fractions had no common component. Although we hypothesized that this lower performance for congruent items would be explained by the use of gap thinking, this turned out not to be the case: evidence was insufficient to show that the applicability of the gap thinking strategy modulated either participants’ accuracy rates or response times (although individual-level data suggest that there is an effect for response times). When fractions shared a common component, instead, our data display a more complex pattern that expected: an advantage for congruent items is present in the first experimental block but fades as the experiment progresses. Numerical distance had an effect in fraction comparison that was statistically significant for items without common components only. Altogether, our results from experts’ reasoning reveal nuances in the fraction comparison task with respect to previous studies and contribute to future models of reasoning in this task.

## Introduction

Rational numbers are key content in mathematics curricula throughout the world. They are usually taught after natural numbers and constitute students’ first approach to concepts such as non-whole quantities and dense sets (Vamvakoussi and Vosniadou, 2004). Learning rational numbers involves understanding multiple aspects such as rational number magnitude and novel algorithms for the arithmetic operations. Successful learning of some of these aspects (e.g., the notion of numerical magnitude) has been shown not only to correlate with future math achievement (Booth and Newton, 2012; Siegler et al., 2012; Booth et al., 2014; Torbeyns et al., 2015) but also to be linked to performance in a diversity of jobs (McCloskey, 2007; Handel, 2016; Liu and Fernandez, 2018) as well as to health outcomes and perception of health risks (Reyna and Brainerd, 2007). Therefore, it is not surprising that an increasing number of studies are focusing on the learning and understanding of fractions, within mathematics education (e.g., Cramer et al., 2019; Flores et al., 2019; Olfos and Rodríguez, 2019; Reinhold et al., 2020) as well as cognitive psychology and neuroscience (e.g., Avgerinou and Tolmie, 2019; Rossi et al., 2019; Stelzer et al., 2019; Cui et al., 2020).

### Difficulties in the Learning of Rational Numbers

Fractions typically constitute the entrance point to the learning of rational numbers through the concept of parts of a whole, although rationals can be learned, and conceptualized in several other different ways (Kieren, 1976). Many studies attest to the difficulty that school children (e.g., Reys et al., 1982; Witherspoon, 2019) and even teachers (e.g., Depaepe et al., 2015) face in order to understand fractions and rational numbers in a mathematically and pedagogically mature manner. This conceptual diversity poses a major challenge to the scientific study of the cognitive processing of fractions (e.g., Bonato et al., 2007; Ischebeck et al., 2009; Schneider and Siegler, 2010; Gabriel et al., 2013a; Barraza et al., 2014).

Several early studies on the cognitive processing of fractions asked whether these numbers are mentally represented as approximate magnitudes (e.g., 1/2 as a magnitude located midway between 0 and 1 on a mental number line) or as pairs of natural numbers (e.g., 1/2 as the pair {1,2}, Bonato et al., 2007; Meert et al., 2010; Schneider and Siegler, 2010; Sprute and Temple, 2011). These studies, together with others in the field of numerical cognition (e.g., Kallai and Tzelgov, 2009; Gabriel et al., 2013b), suggest that fractions are not cognitively processed in an automatic manner, in sharp contrast with natural numbers (Moyer and Landauer, 1967; Henik and Tzelgov, 1982). A possible explanation of this difference is the fact that the standard notations for rational numbers and fractions include natural numbers within them, like a numerator, and a denominator. Kallai and Tzelgov (2012) suggested that the automatic processing of these natural number components hinders the ability to process automatically the fractions themselves.

Many school children and adults fail to recognize fractions as numbers that have a magnitude of their own, whether because of this increased complexity in cognitive processing or due to the lack of an adequate conceptual foundation (Ni and Zhou, 2005). This failure prevents them from working with fractions as holistic entities and allows them to use only strategies based on the fraction components, leading students to think about fractions in a naïve way as if their natural number components were isolated numbers. Many school children think, for instance, that 3/7 > 3/5 because 7 is greater than 5, or that 7/8 + 12/13 is approximately 19 or 21 (Reys et al., 1982; Ni and Zhou, 2005). This naïve generalization from natural to rational number knowledge has been taken as an indication that natural number knowledge interferes with the learning of rational numbers (Hartnett and Gelman, 1998).

Several researchers have proposed that a conceptual reorganization is needed to learn fractions and rational numbers successfully (Vamvakoussi and Vosniadou, 2004; Gabriel et al., 2013a; Van Hoof et al., 2017), a process that often generates cognitive conflict between the previous natural number’s, and the novel rational number’s knowledge (Van Dooren et al., 2015). The difficulties arising from the lack of such reorganization have been extensively investigated in the last decade, being often attributed to a whole number bias, or natural number bias (Ni and Zhou, 2005; Vamvakoussi et al., 2012; Van Hoof et al., 2013). Highly intuitive concepts from the natural number domain that do not exist in the rational number domain, such as successors and antecessors, may also contribute to this issue (Izard et al., 2008). Conversely, there are notions that are unique to the set of rational numbers, such as density (the fact that between any two given rational numbers, there are infinitely many other rational numbers). Rational number density is a particularly difficult concept to master (Vamvakoussi et al., 2012; McMullen et al., 2015; McMullen and Van Hoof, 2020), as it represents the logical opposite of the existence of antecessors and successors. In line with this, many mistakes made by school children relate to the incorrect application of the concepts of successor and antecessor to rational numbers, such as believing that between 1/5 and 4/5, there are only two other rationals (Vamvakoussi and Vosniadou, 2004).

Yet another issue contributing to the difficulty of learning fractions is that the numerical magnitude of a fraction is in some sense independent of the specific components of the fraction, since any fraction can be written in many different but equivalent forms (e.g., 2/3, 4/6, or 6/9). Children who view fractions as no more than a pair of natural numbers might particularly struggle with the notions of fraction equivalence and magnitude (Pearn and Stephens, 2004; Stafylidou and Vosniadou, 2004). On the other hand, people with a deep knowledge of fractions and rational numbers are able to focus on fraction magnitude beyond the fractions’ components (Obersteiner et al., 2013; DeWolf and Vosniadou, 2015).

### Experts’ Performance

Performance in fraction comparison, as well as its possible relation to a natural number bias, has been investigated in populations displaying a variety of knowledge level: middle school students (Van Hoof et al., 2013; Gómez and Dartnell, 2019), mathematics teachers (Siegler and Lortie-Forgues, 2015), and experts (Bonato et al., 2007; Obersteiner et al., 2013; DeWolf and Vosniadou, 2015). Investigating the performance of mathematics experts when comparing fractions allows us to observe an advanced stage in the mathematics development process, informing us about the mature state of fraction knowledge.

Several tests have been proposed to measure knowledge related to fractions, mostly in school children populations (e.g., Gabriel et al., 2013a; Van Hoof et al., 2015). Whether these tests constitute a measure of expertise with fractions–beyond mere knowledge–applicable to both children and adults is, however, an open question. One way of studying fractions with expert populations is to work with undergraduate/graduate students of programs with high mathematical demands, who can be reasonably expected to be experts in elementary school mathematics. Early studies on fraction comparison such as Bonato et al. (2007) resorted to undergraduate students of engineering and physics. Obersteiner et al. (2013), instead, worked with professional mathematicians with a focus on investigating the emergence of a natural number bias in a fraction comparison task. They recruited professors, PhD students, and postdocs from mathematics, applied mathematics, and computer science departments.

The fraction comparison items used by Obersteiner et al. (2013) were categorized by means of two main dimensions: the presence or absence of a common component between the fractions, and the congruency relation between the mathematically correct answers and the answers expected from the application of a natural number bias. A fraction comparison item was called *congruent* if the numerically larger fraction had the larger numerator and denominator, *incongruent* if the numerically larger fraction had the smaller numerator and denominator, or *neutral* otherwise (see examples in Table 1). Results were consistent with a natural number bias in the experts’ answers when presented with fractions with a common component, as congruent items were answered more quickly than incongruent items. In contrast, comparing fractions that lacked a common component led to conflicting results, as congruent items were answered more slowly than incongruent ones. Although both differences in response times (RTs) were statistically significant, the one for items without common components vanished after the removal of a subset of “easy” items that affected mainly the set of congruent items (Obersteiner et al., 2013). This raises the question of whether the observed reversal of the congruency effect is an essential feature of experts’ fraction comparison or if it was due to the particular fraction comparison items chosen. Favoring the former option, a reanalysis of a group of fraction comparison datasets (Gómez and Dartnell, 2015) suggested that this reversed congruency effect is not an isolated finding.

**TABLE 1:** Item types and examples from the fraction comparison task.

Components	Congruency	Gap thinking	# of items	Example
With a common component	Congruent	Leads to the correct answer	36 (12 per distance)	$\frac{31}{84}vs.\frac{17}{84}$ (0.167)
	Incongruent	Leads to the correct answer	36 (12 per distance)	$\frac{16}{35}vs.\frac{16}{75}$ (0.244)
Without common components	Congruent	Leads to the correct answer	12 (4 per distance)	$\frac{37}{55}vs.\frac{68}{81}$ (0.167)
		Leads to the incorrect answer	12 (4 per distance)	$\frac{59}{79}vs.\frac{16}{31}$ (0.231)
		Both fractions have the same gap	12 (4 per distance)	$\frac{45}{64}vs.\frac{75}{94}$ (0.095)
	Incongruent	Leads to the correct answer	36 (12 per distance)	$\frac{62}{97}vs.\frac{55}{69}$ (0.158)
	Neutral	Leads to the correct answer	36 (12 per distance)	$\frac{67}{87}vs.\frac{52}{97}$ (0.234)

*The task included 180 items, split into seven types. Each item type included items with three different numerical distances between the fractions. For each example, the numerical distance between the fractions is shown in parentheses.*

Bonato et al. (2007) provided data from expert and non-expert undergraduates, suggesting that the numerical distance between fractions played no role in comparative judgments of fractions. However, this interpretation has been called into question by later research (e.g., Schneider and Siegler, 2010; Sprute and Temple, 2011). Gabriel et al. (2013b) showed that adults may access fractions’ numerical magnitude depending on the task. Considering specifically math experts, another important study is that of Obersteiner et al. (2013), who aimed at understanding the role of congruency and numerical distance in fraction comparison in such a population. Their study, however, had two shortcomings. First, interpreting mathematical expertise as professional experience in academia strongly restricts the population under study. This leads to difficulties in recruiting participants but, more importantly, to questioning the extent to which the empirical results of such a specialized population generalize to broader populations (see Cipora et al., 2016, for an example where experts’ mathematical performance differs from the broader population). In this sense, the consideration of undergraduate or graduate students of programs with high mathematical demands holds interest.

Studying such a sample (undergraduates from a mathematics department), DeWolf and Vosniadou (2015) found a reversed congruency effect similar to the one reported by Obersteiner et al. (2013). Gómez and Dartnell (2019) presented a fraction comparison task to a large sample of middle school children and observed an association between high general mathematics achievement and a reversed congruency effect as well. Although these works have shown that mathematical expertise tends to be associated with a reversal of the congruency effect for fraction pairs with no common component, no satisfactory explanation for this association has been given.

A second issue relates to the way in which items are presented. Obersteiner et al. (2013) presented in separate blocks items with a common component and items without common components, potentially allowing experts to adapt their strategies for each of these item types. An alternative to blocked presentation is mixing item types within blocks, a manipulation shown to affect test outcomes in several experimental paradigms (e.g., Los, 1996; Odic et al., 2014). DeWolf and Vosniadou (2015) did not consider items with a common component in their design, and it is uncertain if Gómez and Dartnell (2019) results extend to adult populations. It is thus an open question if Obersteiner et al.’s (2013) results, particularly congruency effects, will be replicated when items with and without common components are presented in a mixed manner, as this manipulation may strongly influence participants’ strategy choices.

### Strategies in Fraction Comparison

As mentioned above, the cognitive processing of fractions is not automatic (Kallai and Tzelgov, 2009; Gabriel et al., 2013b). Further evidence for this comes from the range of average RTs of fraction comparison tasks, within seconds (e.g., Bonato et al., 2007; Schneider and Siegler, 2010; Sprute and Temple, 2011; Vamvakoussi et al., 2012; Obersteiner et al., 2013). Therefore, fraction comparison is a task in which the use of strategies is highly relevant. Several works have observed or inferred variability both across and within participants in terms of the strategies that are used to solve the task (Pearn and Stephens, 2004; Clarke and Roche, 2009; Gómez and Dartnell, 2019). It is likely that people with a high level of mathematics expertise use a broad diversity of strategies to compare fractions, opening the question of whether some of these strategies could drive the reversal of the congruency effect.

Throughout this work, we understand strategies in the sense used by Siegler and Araya (2005), as “any goal-oriented, non-obligatory procedure, rather than in the more restricted sense of a conscious, rationally chosen procedure” (p. 3). An individual typically chooses strategies from a dynamic pool, based on specific item characteristics as well as the past performance associated to each strategy (Siegler, 1996; Siegler and Araya, 2005). In this sense, there are many strategies that students use to compare fractions. Some are explicitly taught at school, whereas others are spontaneously devised by them. Examples include benchmarking, which uses a well-known fraction magnitude as an anchor (e.g., 5/7 is larger than 3/8 because the latter is smaller than 1/2 while the former is larger than 1/2; Clarke and Roche, 2009; González-Forte et al., 2018; Obersteiner et al., 2020); naïve componential strategies such as the ones identified by Ni and Zhou (2005), where a fraction is deemed large if its components are large (see also Stafylidou and Vosniadou, 2004); and gap thinking (Pearn and Stephens, 2004; Mitchell and Horne, 2010, 2011; Barnett, 2016; Fagan et al., 2016), where a fraction is deemed large if the difference between its numerator and denominator (its *gap*) is small. Table 2 presents examples of fraction comparison items that are answered correctly or incorrectly for each of these three strategies, together with prototypical examples of reasoning.

**TABLE 2:** Example items and prototypical explicit reasoning corresponding to three possible strategies to compare fractions. Example items taken from Gómez and Dartnell (2019).

	Congruency	Gap thinking	Benchmark to 1/2
Brief description	“Larger components, larger fraction”	“Smaller gap, larger fraction”	
Example item that the strategy answers correctly	17/19 *vs*. 4/9	5/16 *vs*. 12/17	6/14 *vs*. 6/8
Example item that the strategy answers incorrectly	6/13 *vs*. 4/5	10/17 *vs*. 3/9	–
Prototypical explicit reasoning	*“17 is larger than 4, and 19 is larger than 9, so 17/19 must be larger than 4/9”*	*“5/16 is missing 11 pieces to complete the whole, while 12/17 is missing only 5 pieces; therefore, 12/17 must be larger than 5/16”*	*”6/14 is smaller than 1/2, and 6/8 is larger than 1/2; therefore, 6/8 must be larger than 6/14”*

Of particular interest to us is gap thinking, as it turns out to be a mathematically incorrect strategy (because it disregards the size of the parts missing to complete the whole) that leads to correct answers in a very large subset of the set of all possible fraction comparison items. For instance, in a task presented to expert mathematicians (Obersteiner et al., 2013), 84 out of 90 items would have been answered correctly by this strategy. In two of the remaining items, the fractions to be compared had the same gap, and in the other four, the larger fraction of each pair had a larger gap. DeWolf and Vosniadou (2015) presented a fraction comparison task to a group of mathematically skilled undergraduate students, where 24 out of the 30 items that involved proper fractions (those smaller than 1) could be answered correctly by using gap thinking.

Importantly for the understanding of the effect of congruency in the cognitive processing of fraction comparison, all the exceptional items—those for which gap thinking does not lead to the correct answer—belong to the same category: congruent items without common components. This is not due to biases in item selection in previous studies but, rather, due to a mathematical property that can be proved after formally defining the involved concepts (see the Supplementary Material). González-Forte et al. (2019) conducted a clustering analysis with fraction comparison data from primary and secondary school students and observed a cluster of students whose answers aligned with the gap thinking strategy. Interestingly, the size of this cluster represented about 30% of 10th grade students, indicating that gap thinking is used by a relevant proportion of the population. We therefore speculated that the reversed congruency effect for fraction pairs without common components documented in experts’ performance might be due to reliance on the gap thinking strategy.

### The Present Study

The main question guiding this research was whether the conflicting results about congruency effects can be explained in a highly mathematically skilled population by gap thinking, namely, if experts’ lower performance in congruent than in incongruent fraction comparison items without common components can be explained by the use of this strategy. To the best of our knowledge, fraction comparison performance differences due to the gap relation between fractions have been approached from a qualitative perspective in educational research (e.g., Pearn and Stephens, 2004; Clarke and Roche, 2009), whereas congruency effects have been mostly studied from a quantitative perspective, and an approach closer to cognitive psychology (e.g., Obersteiner et al., 2013; Van Hoof et al., 2013). These differences in paradigms and research traditions raise difficulties for integrating the results of both lines of research into a coherent account.

In this work, we designed a fraction comparison task allowing us to analyze and contrast congruency and gap effects within a population of mathematical experts. Our study followed a similar methodology to Obersteiner et al.’s (2013) work, by presenting a computerized fraction comparison task to undergraduate engineering students of a highly selective faculty, including items that allowed us to disentangle those effects. Differently from that study, we used mixed rather than blocked ordering for the presentation of items in order to hinder participants’ reliance on strategies that are specifically tailored for items with or without common components.

As a secondary research question, we tested the role of the numerical distance between fractions in predicting participants’ RTs after eliminating the applicability of the 1/2-benchmark strategy, by using in all items only fractions below or above this value.

## Materials and Methods

### Participants

Fifty-seven undergraduate students (39 men, 18 women) of a diversity of engineering programs participated in this study. The age of one participant was not recorded because of experimenter error. The others had an average age of 21.1 years (SD = 2.2, range = 19–31). All participants were recruited in the Faculty of Physical and Mathematical Sciences of Universidad de Chile (Santiago, Chile), one of the most selective schools of engineering in the country according to the national university selection tests.

The protocols of this research (part of a larger project, Fondecyt 1160188) were reviewed and approved by the Ethics Committee of the Faculty of Medicine of Universidad de Chile. Accordingly, participants signed an informed consent form and were rewarded with CLP 2,000 regardless of their performance in the task.

### Task Items

Pairs of fractions to be compared were selected according to the following constraints: (a) denominators ranged from 31 to 99; (b) numerators ranged from 11 up to the corresponding denominator minus 11; (c) in each pair, both fractions were on the same side of 1/2 (either both above or both below); (d) whenever fractions had a different numerator and/or denominator, the numerical distance between these was at least 5; and (e) the numerical distance of fraction gaps for fraction pairs with different gaps was at least 5. Constraints (a) and (b) were aimed at discarding simple and common fractions (e.g., those with single-digit numerators and denominators that may be processed differently from other fractions, Liu, 2018) as well as fractions that are too close to 0 or to 1. Constraint (c) was included to avoid participants’ using the benchmarking-to-1/2 strategy. Finally, constraints (d) and (e) were included to have clear differences between items with the same numerator, denominator, or gap, and items with different ones. In addition, to reduce attempts at simplifying reducible fractions, we discarded all those fractions whose numerator and denominator had 2, 3, 5, or 11 as a common factor.

From all the possible fraction pairs fulfilling the above constraints, we randomly selected 180 pairs according to the following classifications: (a) presence/absence of a common numerator or denominator; (b) congruency: congruent, incongruent, or neutral pairs; (c) applicability of gap thinking: pairs in which gap thinking leads to the correct answer or to the incorrect answer, or pairs in which both fractions have the same gap; and (d) numerical distance between the two fractions: small (about 0.10), medium (about 0.17), and large (about 0.24). The number of items selected for each type, together with examples, is presented in Table 1 (the full set of items is included in the Supplementary Material as Supplementary Table S1).

### Procedure

The fraction comparison task was presented in a computerized format, in the computer science classrooms of the faculty. The task was program using Python 2.7 and Pygame 1.9.1. Fraction pairs were presented in three blocks of 60 pairs each, and participants were randomly assigned to one of ten possible item orderings. For each ordering, the different item types were presented in an interleaved manner, and the location of the correct answer (left/right) was randomized taking care that the correct answer was not on the same side of the screen for more than three consecutive items.

Each item started with the presentation of the question “Which of these fractions is LARGER?” (in Spanish) and a fixation dot in the middle of the screen for 500 ms. Then the two fractions were presented side by side, and they remained on the screen until the participant answered using the keys *Q* or *P* (left/right fraction, respectively) or until a time limit of 10 s was reached. If this limit was reached, the item was considered as omitted. A blank screen followed for 1 s before displaying the next item.

### Data Analysis

Statistical analyses were conducted using *R* (64-bit) 3.4.1, running in *R*-Studio 1.1.447. Accuracy rates were computed based on non-omitted items only. Average RTs were computed considering only items that were answered correctly. General data manipulation was done with the help of the package dplyr, version 0.8.0.1 (Wickham et al., 2019).

We used analyses of deviance, a generalization of analysis of variance for generalized linear mixed regression models, for the study of accuracy rates and RTs as a function of diverse fixed factors (e.g., congruency), considering both participants and items as random factors. These generalized linear mixed models were analyzed using the package lme4, version 1.1–13 (Bates et al., 2015), and the fixed factors’ statistical significance was assessed using Wald’s X2 type III tests as implemented in the package car, version 2.1–5 (Fox and Weisberg, 2011). The statistical significance of regression coefficients for the analysis of the effect of numerical distance on RTs was assessed using Satterthwaite approximations of the tests’ degrees of freedom as implemented in the package lmerTest, version 2.0–33 (Kuznetsova et al., 2017).

The raw data analyzed in this manuscript, as well as the analysis script, are available as Supplementary Material.

## Results

We excluded from the analysis one participant who omitted 24 out of the 180 items (13.3%). The remaining 56 participants omitted, on average, 2.3 items, or 1.3% of the total (SD = 1.6%, range = 0%–6.1%).

### Overall Performance

Table 3 presents descriptive statistics for accuracy and RTs. For both measures, performance was significantly better when fractions shared a common component [accuracy (acc): 98% *vs*. 91%, *t*(55) = 10.0, *p* < 0.0001; RT: 2,795 ms *vs*. 4,009 ms, *t*(55) = 14.6, *p* < 0.0001].

**TABLE 3:** Overall performance in the fraction comparison task. Standard deviations in parentheses.

Items	Accuracy	Response time (ms)
With a common component	98% (2%)	2,795 (739)
Without common components	91% (5%)	4,009 (993)
Total	94% (3%)	3,494 (829)

**FIGURE 1:**
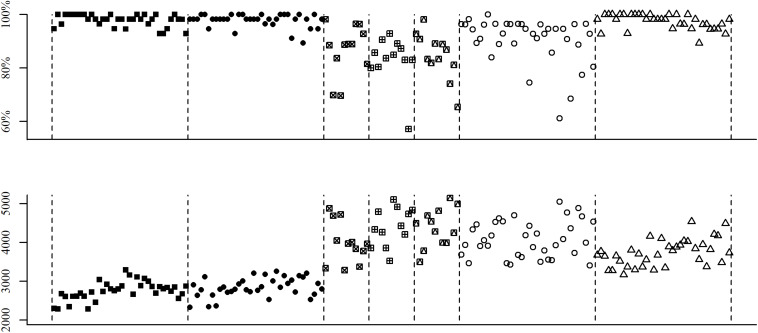
Accuracy rates and response times (RTs) for all items. Mean accuracies (top) and RTs (bottom) separately for each item of the fraction comparison task. Different shapes represent different item types, from left to right: congruent with a common component, incongruent with a common component, congruent without common component (gap thinking leads to correct answer), congruent without common component (gap thinking leads to incorrect answer), congruent without common component (both fractions have the same gap), incongruent items without common components, and neutral items without common components.

We also looked at item-level variability, in order to understand the degree of consistency between participants’ performance for different items within each category. Figure 1 depicts average accuracy rates and RTs for all items, revealing that some item types displayed a much higher variability than others. We quantified this dispersion by computing median absolute deviations (MADs; a robust alternative to SDs) for each item type. Overall, items with a common component showed the lowest MADs, whereas congruent and incongruent items without common components exhibited the highest MADs, with neutral items in between (see Table 4).

**TABLE 4:** Median absolute deviations (MADs) for all items within each type.

Components	Congruency	Gap thinking	Accuracy MADs	Response time MADs
With a common component	Congruent	Leads to the correct answer	2.7%	196
	Incongruent	Leads to the correct answer	2.7%	245
Without common components	Congruent	Leads to the correct answer	9.2%	584
		Leads to the incorrect answer	5.1%	639
		Both fractions have the same gap	5.9%	583
	Incongruent	Leads to the correct answer	4.2%	601
	Neutral	Leads to the correct answer	2.7%	343

Although it is not the focus of the present work to contrast our findings with a non-expert group, as part of the larger project in which this research took place, we also gathered data from such a population. The average accuracy rate in this non-expert sample was 76% (SD = 22%; data not published), supporting the consideration of engineering students as an expert sample.

### Congruency Effects

We computed average accuracies and RTs for each of the item types defined by components and congruency (see Table 5). Interesting contrasts emerged when comparing our data with those of Obersteiner et al.’s (2013) mathematicians. Our participants were about 10% less accurate when responding to congruent items without common components and about 800–900 ms slower when responding to items with a common component. This pattern of differences may be partly explained by item presentation: Obersteiner et al. (2013) presented items with and without common components in separate blocks, allowing mathematicians to apply componential strategies that are tailored for items with a common component, allowing them to answer these items more quickly.

**TABLE 5:** Performance by components and congruency.

Components	Congruency	Accuracy	Response time (ms)
With a common component	Congruent	98%(3%)	2,752(787)
	Incongruent	98%(2%)	2,839(739)
Without common components	Congruent	85%(13%)	4,292(1,217)
	Incongruent	91%(7%)	4,058(1,012)
	Neutral	97%(3%)	3,747(934)

*Mean accuracies and response times (RTs) for each of the item types defined by components and congruency. Standard deviations in parentheses.*

The statistical analysis of accuracy rates revealed a significant interaction between components and congruency [*X*^2^(1) = 3.9, *p* = 0.05]. This interaction reflects the fact that congruent and incongruent items were answered with similar accuracy when items shared a common component [*t*(55) = 0.2, *p* = 0.83], but congruent items without common components were answered less correctly than their incongruent counterparts [*t*(55) = 2.9, *p* = 0.005]. A similar analysis for RTs showed a significant interaction as well [*X*^2^(1) = 5.7, *p* = 0.02], stemming from a non-significant advantage for congruent items when items share a common component [*t*(55) = 1.7, *p* = 0.09], and a significant advantage for incongruent items when items lack common components [*t*(55) = 3.2, *p* = 0.002].

Looking at the individual level, we observed that 16 participants obtained higher accuracies in congruent than in incongruent items with a common component, whereas 12 participants showed the opposite pattern. In contrast, the relation for items without common components reversed to 21 *vs*. 32 participants. As for RTs in the common component case, 35 participants exhibited quicker responses to congruent items and 21 participants quicker responses to incongruent items, whereas in the non-common component case, this relation reverses to 19 *vs*. 37 participants. Fisher’s exact tests for count data indicated that the reversal for accuracy rates was not significant (odds ratio = 2.01, *p* = 0.16), while that of RTs was significant (odds ratio = 3.21, *p* = 0.004).

The analysis of neutral items, compared to the other items without common components, showed significantly greater accuracy rates and smaller RTs than both congruent [acc: *t*(55) = 7.8, *p* < 0.0001; RT: *t*(55) = 6.0, *p* < 0.0001] and incongruent [acc: *t*(55) = 7.7, *p* < 0.0001; RT: *t*(55) = 5.1, *p* < 0.0001] items.

**FIGURE 2:**
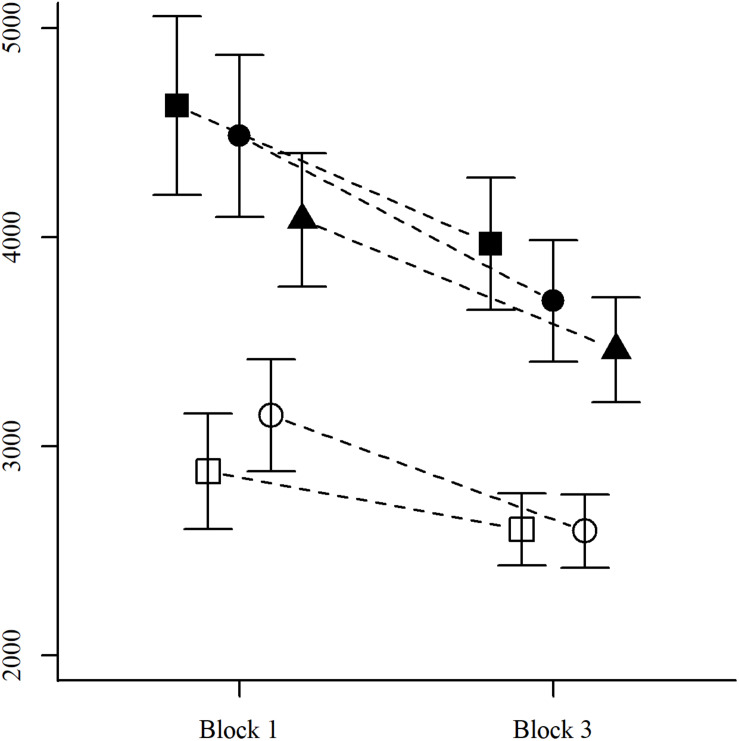
Response times by components, congruency, and experimental block. Mean RTs for each of the item types defined by components (white: with a common component, black: without) and congruency (■: congruent, •: incongruent, and ▲: neutral), separately for items in the first and last experimental blocks. Vertical bars depict 95% confidence intervals.

In contrast with previous studies (Vamvakoussi et al., 2012; Obersteiner et al., 2013), our RT data showed a non-significant difference between congruent and incongruent items with a common component. Given that this result has consistently emerged in the past, we conducted a *post hoc* exploration by looking at items with a common component presented during the first and last blocks of the testing session (each block contained 60 items in total; see Supplementary Table S2 in the Supplementary Material). Whereas accuracies showed no relevant changes between these blocks, RTs for items with a common component displayed a significant interaction between block and congruency [*X*^2^(1) = 11.7, *p* = 0.0006], reflecting the presence of a significant advantage for congruent items in the first experimental block [*t*(55) = 3.2, *p* = 0.002] but not in the last block [*t*(55) = 0.15, *p* = 0.88; see Figure 2].

Regarding RTs for items without common components, our data showed the expected reversed congruency effect: congruent items were answered more slowly than incongruent items (see also Obersteiner et al., 2013; DeWolf and Vosniadou, 2015). A *post hoc* exploration comparing the first and last blocks of items suggests that this difference remained unchanged throughout the experimental session, as there was no significant interaction between block and congruency [*X*^2^(2) = 0.9, *p* = 0.64].

### Congruency Effects and Gap Thinking

Obersteiner et al. (2013) suggested that the reversed congruency effect for items without common components stemmed from specific items, particularly those where one fraction had a numerator 1 or 2 units smaller than its corresponding denominator (i.e., where one of the fractions had a gap of 1 or 2). After discarding these items, the reversed congruency effect they observed was no longer statistically significant. Our item set was chosen so that no fraction had a gap smaller than 11 [see constraint (b) in the section “Task Items”], allowing us to exclude this alternative explanation, but still our data revealed a significant reversed congruency effect. To dig into a possible cause of this difference, we asked whether the applicability of the gap thinking strategy made a difference in participants’ responses, by computing accuracy rates and RTs for congruent items without common components separately for those in which gap thinking leads to the correct answer and to the incorrect answer, and for items in which both fractions have the same gap (see Table 6). We remind the reader that these three possibilities can only occur in the case of congruent items without common components, while for all the other item types, gap thinking always leads to the correct answer.

**TABLE 6:** Performance by gap type.

Item type	Accuracy	Response time (ms)
Gap thinking leads to the correct answer	87% (11%)	4,021 (1,255)
Gap thinking leads to the incorrect answer	83% (18%)	4,483 (1,427)
Both fractions have the same gap	84% (16%)	4,417 (1,296)

*Mean accuracies and RTs for congruent items without common components, separately for each type defined by the applicability of gap thinking. Standard deviations in parentheses.*

Against our hypothesis, the applicability of gap thinking had no significant effect on either participants’ accuracy rates [*X*^2^(2) = 1.7, *p* = 0.44] or RTs [*X*^2^(2) = 5.0, *p* = 0.08]. A *post hoc* analysis of RTs showed that participants took significantly less time to answer items where gap thinking leads to the correct answer (i.e., where the larger fraction has the smaller gap) with respect to items where gap thinking leads to the incorrect answer [*t*(55) = 3.7, *p* = 0.0005] or to items where both fractions share the same gap [*t*(55) = 3.6, *p* = 0.0006], whereas the latter two item types did not statistically differ from one another [*t*(55) = 0.6, *p* = 0.56]. The lack of statistical significance of gap in the linear mixed model for RTs, as it includes random factors, suggested the presence of a high variability in RTs across items within the gap item types. This was confirmed by computing the intraclass correlation coefficient, *ICC* = 0.11, indicating a very low similarity of RTs within each type.

Looking at the individual level, the relation between participants who had better accuracy in items where gap thinking leads to the correct *vs*. to the incorrect answer and *vice versa* was 28:18 (*p* = 0.18, binomial test). For RTs, this relation was 38:18 (*p* = 0.01). The corresponding figures for the contrast between items where gap thinking leads to the correct answer vs. items in which gap thinking is uninformative are 22:20 (accuracies, *p* = 0.88) and 40:16 (RTs, *p* = 0.002).

As a final *post hoc* analysis, we regressed RTs on the distance between fractional gaps for each item type, leading to no significant effects [items where gap thinking leads to correct answer: *b* = −63, *SE* = 29, *t*(9.8) = −2.2, *p* = 0.06; items where gap thinking leads to the incorrect answer: *b* = 1, *SE* = 21, *t*(10.1) = 0.1, *p* = 0.95].

### Numerical Distance

**FIGURE 3:**
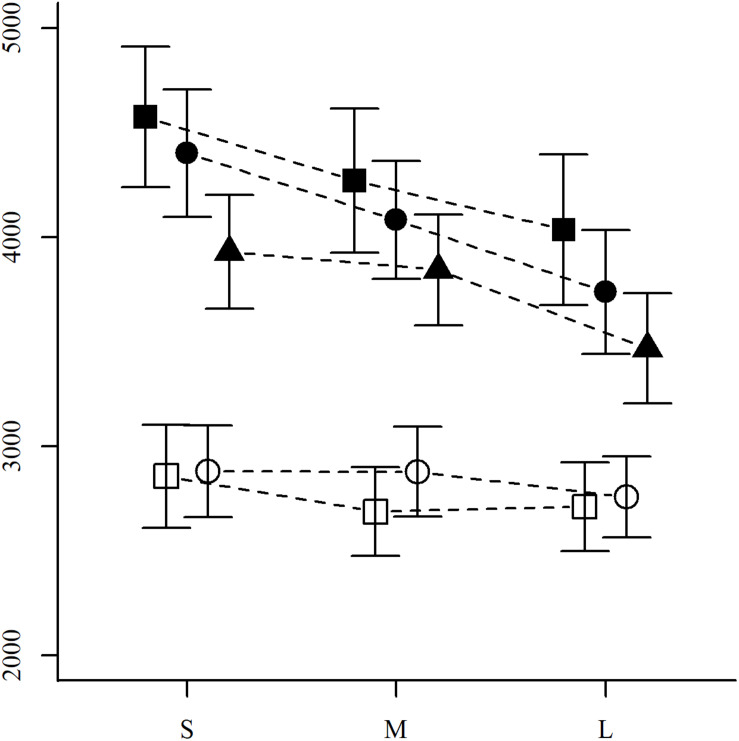
Response times by components, congruency, and numerical distance. Mean RTs for each of the item types defined by components (white: with a common component, black: without) and congruency (■: congruent, •: incongruent, and ▲: neutral), separately for each of the three possible numerical distances between fractions (small, medium, and large). Vertical bars depict 95% confidence intervals.

As a final analysis, we investigated the effect of numerical distance on participants’ RTs. Since all our items were chosen so that both fractions were either above or below 1/2, our test of this effect is less affected than previous studies by participants’ use of strategies such as benchmarking against 1/2. We computed average RTs for each level of numerical distance present in our item set, categorized into small, medium, and large distances (approx. 0.10, 0.17, and 0.24, respectively; see Table 1 for examples). Overall, there is a significant effect of numerical distance [*X*^2^(1) = 8.8, *p* = 0.003; RTs for items with small, medium, and large distances were 3,655, 3,512, and 3,303 ms, respectively]. However, as Figure 3 shows, this effect was markedly different for items with and without common components. Table 7 presents the results of linear regressions applied separately to each combination of components and congruency, showing that although RTs for all item types tend to decrease with increasing numerical distance, this change is only statistically significant for items without common components.

**TABLE 7:** Response time regressions for fractional numerical distance, by components and congruency.

Components	Congruency	*b*	*t*	d.f.	*p*
With a common component	Congruent	−1,031(680)	–1.5	33.9	0.14
	Incongruent	−879(715)	–1.2	34.0	0.23
Without common components	Congruent	−4,149(1,416)	–2.9	34.2	0.006
	Incongruent	−4,992(1,066)	–4.7	34.4	< 0.0001
	Neutral	−3,281(865)	–4.0	33.5	0.0006

*Results of linear mixed regressions applied to RTs, considering the numerical distance between fractions as a fixed factor and participants and items as random factors.*

Following previous studies (Bonato et al., 2007; Obersteiner et al., 2013), we also looked at the effect of fractional numerical distance as opposed to that of the distances between fraction components (numerators and denominators). Table 8 presents the outcome of multiple regressions for RTs of items without common components, considering numerator and denominator distances in addition to fractional numerical distance. The only case in which numerical distance explained RTs above and beyond componential numerical distance was that of incongruent items. For neutral items, in contrast, statistical significance was reached for the denominator distance only. This suggests that for these items, the effect of numerical distance on RTs is mediated by the denominator distance.

**TABLE 8:** Response time regressions for fractional and componential numerical distance, by congruency.

Congruency	Predictor	*b*	*t*	d.f.	*p*
Congruent	Frac	−2,679(1,666)	–1.6	31.7	0.12
	Num	−26.8(14.7)	–1.8	31.1	0.08
	Den	18.6 (9.6)	1.9	31.1	0.06
Incongruent	Frac	−4,198(1,261)	–3.3	31.7	0.002
	Num	18.5 (13.1)	1.4	31.8	0.17
	Den	−5.6(8.1)	–0.7	31.4	0.50
Neutral	Frac	−835(1,298)	–0.6	31.3	0.52
	Num	−26.0(18.5)	–1.4	31.5	0.17
	Den	−20.7(8.0)	–2.6	31.2	0.01

*Results of linear mixed regressions applied to RTs of items without common components, considering the numerical distance between fractions (Frac) as well as the numerical distance between fraction components (Num, Den) as fixed factors and participants and items as random factors.*

## Discussion

We presented a fraction comparison task to a sample of undergraduate engineering students to test the role of different item characteristics and strategies in their performance. We measured accuracy rates and RTs to a set of fraction comparison items that were categorized in terms of the presence of a common component, congruency, the applicability of gap thinking, and numerical distance. Our data aligned in some respects with previously reported results but also revealed interesting nuances.

### Variability at the Individual and Item Levels

Our data show important variability at both the individual and the item levels. Particularly regarding response accuracies, variability within item types seems much larger than in other fraction comparison studies such as the one with middle school children by Gómez and Dartnell (2019; see Figure 1 therein). While variability at the individual level has traditionally been considered in statistical inference, variability at the item level has not been fully acknowledged until more recently (Baayen et al., 2008; Bates et al., 2015). Mixed-effects regression models allow researchers to consider the variability that stems from the random selection of participants and of items simultaneously, by means of random factors. In our statistical analyses of congruency, gap, and numerical distance effects, the inclusion of random factors for items allowed us to assess whether our effects of interest were robust enough so as to be generalizable to novel items from the same item population. While the statistical significance of the majority of our results was unaffected by the inclusion of these random factors, it is worth commenting that the gap effect reported in section “Congruency effects and gap thinking,” was significant without the item random factor but non-significant with it. We interpret this change in results between both analyses as revealing that, despite the presence of a significant gap effect within our specific item set, there is not enough evidence that this effect may generalize to novel, similarly constructed items. Alternatively, it is also possible that gap thinking is not consistently used across items. Nonetheless, our results suggest that outcomes like the reversed congruency effect for items without common components can be expected to generalize to novel items.

### The Presence of Common Components and Strategy Selection

Obersteiner et al. (2013) presented items with and without common components in different blocks (see also Vamvakoussi et al., 2012), whereas we intermixed all item types within blocks. We expected mixed ordering to reduce participants’ reliance on strategies that are specific for items with a common component. Although participants in our study took longer overall to answer common component items than in the previous studies, they performed significantly better than in items without common components. This indicates that these highly skilled participants can flexibly adapt and use component-based strategies within the time frame allowed in our design.

### Congruency Effects

As in many previous studies, congruent items with a common component tended to be easier than the corresponding incongruent items (Vamvakoussi et al., 2012; Obersteiner et al., 2013; Gómez and Dartnell, 2015, 2019). This difference, however, was not statistically significant in our data. A more careful analysis, considering the degree of progress of the experimental session, showed that this congruency effect was present in the first block (items 1–60) but became negligible in the last one (items 121–180), indicating that the advantage for congruent items depends on the participants’ level of practice with the task, and/or on task-specific strategies that participants may develop throughout the experimental session.

In the case of pairs where fractions lack a common component, our results confirmed the presence of a reversed congruency effect by showing that congruent items were answered less correctly and more slowly than incongruent items (DeWolf and Vosniadou, 2015; Gómez and Dartnell, 2015, 2019). Individual-level data confirmed this reversed pattern. Whereas in Obersteiner et al.’s (2013) study, this reversed effect could have been explained by the presence of a subset of “easy” items where one of the fractions had a very small gap, this confound does not apply to our set of items. This provides further evidence that the reversed congruency effect is a robust finding that needs to be considered in theories about the cognitive processing of fraction comparison in the context of highly skilled individuals.

It is also relevant to notice that neutral items turned out to be easier (in terms of both accuracy and RT) than both congruent and incongruent items, an outcome that has also been reported previously (e.g., Obersteiner et al., 2013) but not discussed in depth so far. Our analysis of numerical distance effects suggests that this advantage follows from a component-based strategy used specifically for neutral items, as the effect of fractional numerical distance on RTs for these items was modulated by that of denominator distance.

### Congruency Effects and Gap Thinking

As a possible explanation to the reversed congruency effect observed in items without common components, we investigated the role of gap thinking in young experts’ mental processes during fraction comparison. Although mathematically incorrect, gap thinking leads to the correct answer in a very high proportion of cases: out of the 1,101,230 possible fraction pairs that fulfilled the constraints described in section “Materials and Methods,” 84% are answered correctly by gap thinking. Moreover, all items that fail to be answered correctly by this strategy are categorized into one of our main item types: congruent items without common components. Therefore, while it is unclear if it is representative of the fraction comparison items that students are actually presented with in the classroom, gap thinking needs to be explicitly considered in the cognitive study of fraction comparison.

Our data showed that accuracy rate variations due to the applicability of gap thinking were minimal and not statistically significant. RTs, on the other hand, seemed to be affected by gap type, but this effect did not reach statistical significance. Pairwise *post hoc* comparisons suggested, however, that there are significant differences between items in which gap thinking leads to the correct answer and the other two types, implying that the lack of significance of the omnibus test could be a matter of statistical power (due, for instance, to the low intraclass correlation found in RTs across gap types).

An absence of a gap effect both in accuracy rates and in RTs (or a very low effect size) would indicate that, against our expectations, the impaired performance in congruent items without common components is not due to participants using gap thinking. This is consistent with the negative result showing that RTs were not modulated by the numerical distance between fractional gaps. It is possible, nonetheless, that participants do not use gap thinking frequently or consistently enough for gap effects to reach statistical significance (it is worth noting that individual-level data supported a gap effect). Variability in strategy use is inherent to models such as Siegler’s (1996) overlapping waves model, and we can take it as an indication that young experts do not consider (whether consciously or not) fractional gaps consistently for strategy selection in fraction comparison. A promising line of inquiry for further research is that used by González-Forte et al. (2018) with school children, who complemented a fraction comparison task similar to ours with post-task interviews in order to probe participants’ reliance on the gap thinking strategy.

### Numerical Distance

Our data also replicated previously documented numerical distance effects (Schneider and Siegler, 2010; Sprute and Temple, 2011; Obersteiner et al., 2013; DeWolf and Vosniadou, 2015), by showing that items in which fractions are numerically farther apart were quicker to answer. In comparison to these previous studies, the set of items used here controlled for a number of characteristics that strengthen our conclusion. Among these characteristics, the most prominent is that all items were chosen to avoid participants’ use of the benchmarking-to-1/2 strategy.

To further investigate the numerical distance effect for items without common components, we also conducted multiple regressions to pit the effect of numerical distance against that of the distances between fraction components. While items without common components all showed an effect of numerical distance when considered as the only predictor, the addition of componential distances into the models led to very different outcomes. For congruent items, all three predictors fell short of statistical significance. For incongruent items, the effect of numerical distance remained significant after the inclusion of componential distances, suggesting that participants’ reliance on numerical distance is more consistent in this item type. In terms of strategy selection, this could be interpreted as a higher activation of strategies related to numerical distance because of a lower activation of component-based strategies. Finally, for neutral items, the significant effect of numerical distance when considered alone was linked to a significant effect of denominator distance in the multiple regression. This indicates that the numerical distance effect is in this case mediated by the denominator distance and may be the reason why neutral items were answered more quickly than the other item types lacking common components.

### The Fraction Comparison Process

Fraction comparison is a complex cognitive process, and while it has been extensively investigated in the mathematics education literature, cognitive psychology research has focused on it only during the past decade. In this period, the effect of several item characteristics, such as the presence/absence of a common component (Meert et al., 2009; Barraza et al., 2014), congruency (Ischebeck et al., 2009; Vamvakoussi et al., 2012; DeWolf and Vosniadou, 2015; Gómez and Dartnell, 2019), and numerical distance (Bonato et al., 2007; Schneider and Siegler, 2010; Sprute and Temple, 2011; Obersteiner et al., 2013), have been explored. Nonetheless, research has also shown that item and task characteristics are not enough to explain fraction comparison performance and that individual differences and strategies need to be taken into account (Hallett et al., 2010; McMullen et al., 2018; Gómez and Dartnell, 2019). Our research contributes in this direction, investigating in a more systematic way than previous studies one interaction between item characteristics and strategies, specifically that of congruency and gap thinking.

#### The Congruency Account

Congruency has been hypothesized by several researchers as a relevant dimension to understand fraction comparison and other fraction tasks (Ischebeck et al., 2009; Vamvakoussi et al., 2012; Obersteiner et al., 2013; Gómez et al., 2014). However, the evidence for its role in explaining people’s fraction comparison performance is mixed. While congruency is a very strong predictor of school children’s answers in the early stages of learning, where accuracy rates are highly affected by congruency even regardless of the presence of a common component (Gómez et al., 2014; Gómez and Dartnell, 2019), data from skilled individuals and experts have shown a reversed effect (Obersteiner et al., 2013; DeWolf and Vosniadou, 2015; Gómez and Dartnell, 2019). In this study, we confirmed the presence of this reversed effect using a more controlled item set than these previous works and others. This outcome is problematic for the consideration of congruency as a theoretically relevant dimension in experts’ cognitive processes for comparing fractions (see a similar argument by DeWolf and Vosniadou, 2015). While congruency remains correlated with fraction comparison performance when fractions lack common components (i.e., it explains variability in experts’ accuracies and RTs), its conceptual foundation falls apart as it predicts a wrong direction for the effect. Our results might even imply that the congruency effect for fraction pairs with a common component is not as robust as previously expected, as our analysis for the first and third blocks of the experimental session suggests that it may vanish with extended task practice. Altogether, while it is undeniable that congruency correlates with experts’ performance in fraction comparison, it is conceptually unsuccessful in explaining this performance.

As an alternative account for the reversed congruency effect, we explored the role of gap thinking, although results did not align with our expectations. Neither accuracy rates nor RTs were significantly modulated by the applicability of gap thinking, although RTs showed interesting pairwise differences in a *post hoc* analysis as well as in individual-level data. Further research is needed to clarify whether this mixed outcome for RTs is a matter of statistical power or an actual lack of effect. Our data show that the three gap item types have a very low intraclass correlation, indicating that a large share of variance comes from item-specific effects rather than from item types.

Note that even if we considered our RT results as a positive indication of the use of gap thinking, the reduced accuracy for congruent items without common components would remain unexplained. Such reduced accuracies have been documented before, not only with adult experts (DeWolf and Vosniadou, 2015) but also with high-achieving middle schoolers (those in cluster B in Gómez and Dartnell, 2019), showing that this is not an isolated finding and that it needs to be directly addressed in future research.

It is possible that other strategies, not considered in our design, produce this pattern of results. One possibility is a “larger denominator, smaller fraction” strategy (Gómez et al., 2014), which systematically leads to the incorrect answer for congruent items without common components and to the correct answer for all incongruent and neutral items. Additional support for this strategy comes from the significant denominator distance effect we observed for RTs to neutral items. However, the outcomes of this strategy are highly correlated with those of congruency: just like gap thinking, it leads to correct answers for all incongruent and neutral items, but it leads systematically to incorrect answers in congruent items without common components and to no answer for congruent items with a common component (i.e., fraction pairs with the same denominator). It is therefore very difficult to draw conclusions about this strategy based on our item set and data.

#### Numerical Distance Effects

Numerical distance effects have often been considered as evidence of someone’s ability to access the numerical magnitude of fractions (e.g., Bonato et al., 2007; Schneider and Siegler, 2010; Sprute and Temple, 2011). Our data call into question this association, as component-based strategies may also lead to numerical distance effects, adding to the increasing evidence suggesting that working with fraction magnitude is better understood as a process based on strategies rather than on automatic access to a numerical magnitude (Kallai and Tzelgov, 2012; Gabriel et al., 2013b). Recent evidence by Binzak and Hubbard (2020) challenged this account by observing numerical distance effects when allowing participants much shorter time windows to answer each item, a finding that deserves further research for both its theoretical and practical implications.

### Final Remarks

Our study provided fraction comparison data from a highly mathematically skilled population to understand the role of several item characteristics and strategies: congruency, gap thinking, and numerical distance. Results confirm previous outcomes that congruency effects fail to conceptually explain skilled participants’ performance, and also discard a possible alternative explanation represented by gap thinking. Regarding numerical distance in fraction comparison, our study revealed significant effects for items without common components even after controlling for specific strategies such as benchmarking against 1/2. A closer examination, however, reveals a complex pattern where this effect is only robust for one item type, and for another type, it is mediated by denominator distance.

Altogether, this work contributes to our knowledge of the interaction between item characteristics and strategies in the fraction comparison task, helping to unveil the great complexity hidden behind middle school mathematical content. At the same time, our results reveal that item-level variability is important within items without common components, and future work is needed to uncover its sources.

## Data Availability Statement

The full set of data reported in this study and the analysis script are available in the Open Science Foundation servers at https://osf.io/nhtkw/.

## Ethics Statement

The studies involving human participants were reviewed and approved by Comité de Ética de Investigación en Seres Humanos, Facultad de Medicina, Universidad de Chile. The participants provided their written informed consent to participate in this study.

## Author Contributions

PD and DG contributed the conception and design of the study. NM and DG collected the data. NM organized the database. DG performed the statistical analysis. NM wrote the first draft of the manuscript. All authors contributed to manuscript revision and read and approved the submitted version.

## Conflict of Interest

The authors declare that the research was conducted in the absence of any commercial or financial relationships that could be construed as a potential conflict of interest.
