# Supplementary material for: A Study on Congruency Effects and Numerical Distance in Fraction Comparison by Expert Undergraduate Students
Source: Front Psychol. 2020 Jun 18;11:1190. doi: 10.3389/fpsyg.2020.01190 (PMC7315778; doi:10.3389/fpsyg.2020.01190)
Supplement: Supplementary file 1 [file Data_Sheet_1.docx]

Supplementary Material

# Mathematical analysis of the relation between congruency and gap

**Definition 1:** Let $a,b,c,d$ be integers so that $\frac{a}{b}<\frac{c}{d}$. We say that this fraction comparison item is:

- *Congruent* if and only if $a\leq c\wedge b\leq d$.
- *Incongruent* if and only if $a\geq c\wedge b\geq d$.
- *Neutral* if and only if $a<c\wedge b>d$.

**Definition 2:** Let $a,b$ be integers so that $\frac{a}{b}$ is a proper fraction. We define the *gap* of $a,b$ as $gap\left( a,b \right)=b-a$.

**Proposition:** Let $a,b,c,d$ be integers so that $\frac{a}{b}$ and $\frac{c}{d}$ are proper fractions and $\frac{a}{b}<\frac{c}{d}$. Then $gap\left( c,d \right)<gap(a,b)$ unless the comparison item is congruent and the two fractions share no common component.

Proof: Assume that $gap\left( c,d \right)\geq gap(a,b)$. From the inequality $\frac{a}{b}<\frac{c}{d}$, we can obtain that

$$1-\frac{c}{d}<1-\frac{a}{b}$$

and then

$$\frac{gap(c,d)}{d}<\frac{gap(a,b)}{b}$$

Since the two fractions are proper, their gaps are positive and we can rewrite this expression as

$$\frac{gap(c,d)}{gap(a,b)}<\frac{d}{b}$$

Using here the assumption that $gap\left( c,d \right)\geq gap(a,b)$, we conclude that $1\leq\frac{gap(c,d)}{gap(a,b)}<\frac{d}{b}$, and therefore $b<d$.

The inequality $\frac{a}{b}<\frac{c}{d}$ also implies that $\frac{a}{c}<\frac{b}{d}$, and since $\frac{b}{d}<1$ we conclude that $\frac{a}{c}<1$ as well, and therefore $a<c$.

Thus, the fraction comparison item is congruent and it has neither a common denominator nor a common numerator. □

# Supplementary Tables

**Table S1. Full set of items.**

| **Components** | **Congruency** | **Gap** | **Items** | | |
| --- | --- | --- | --- | --- | --- |
| With a common component | Congruent | Leads to the correct answer | 33/65 vs. 49/65  38/99 vs. 46/99  43/88 vs. 21/88  25/82 vs. 17/82  43/99 vs. 25/99  48/91 vs. 69/91  34/55 vs. 39/55  41/85 vs. 19/85  50/79 vs. 63/79  57/86 vs. 49/86  66/91 vs. 80/91  73/88 vs. 63/88 | 11/48 vs. 23/48  71/89 vs. 55/89  47/81 vs. 56/81  23/71 vs. 11/71  43/81 vs. 62/81  26/93 vs. 43/93  54/85 vs. 62/85  51/67 vs. 34/67  29/55 vs. 39/55  29/56 vs. 43/56  43/64 vs. 33/64  16/73 vs. 34/73 | 18/61 vs. 13/61  62/97 vs. 86/97  52/81 vs. 65/81  35/92 vs. 27/92  55/94 vs. 79/94  17/84 vs. 31/84  39/79 vs. 30/79  18/61 vs. 29/61  20/63 vs. 14/63  57/89 vs. 77/89  49/94 vs. 65/94  77/93 vs. 68/93 |
|  | Incongruent | Leads to the correct answer | 25/96 vs. 25/66  16/35 vs. 16/75  31/95 vs. 31/63  38/95 vs. 38/77  14/31 vs. 14/61  46/61 vs. 46/81  11/48 vs. 11/75  13/78 vs. 13/37  21/97 vs. 21/47  22/37 vs. 22/43  45/89 vs. 45/59  67/80 vs. 67/90 | 61/92 vs. 61/72  73/96 vs. 73/86  35/66 vs. 35/46  31/85 vs. 31/64  25/96 vs. 25/58  16/33 vs. 16/63  54/95 vs. 54/79  21/86 vs. 21/49  60/97 vs. 60/71  26/53 vs. 26/79  53/66 vs. 53/95  15/44 vs. 15/83 | 47/71 vs. 47/81  18/89 vs. 18/41  60/79 vs. 60/89  45/86 vs. 45/59  37/52 vs. 37/66  14/97 vs. 14/53  25/52 vs. 25/96  59/71 vs. 59/87  19/48 vs. 19/66  24/73 vs. 24/49  36/71 vs. 36/47  17/36 vs. 17/54 |
| Without common components | Congruent | Leads to the correct answer | 34/65 vs. 57/74  49/65 vs. 76/87  59/70 vs. 23/39  68/81 vs. 37/55 | 29/92 vs. 17/85  83/98 vs. 51/76  23/59 vs. 11/53  64/91 vs. 76/97 | 83/96 vs. 56/89  86/99 vs. 62/81  67/95 vs. 45/83  80/93 vs. 41/66 |
|  |  | Leads to the incorrect answer | 11/31 vs. 43/92  43/95 vs. 20/67  11/43 vs. 38/77  18/55 vs. 41/99 | 16/31 vs. 59/79  36/77 vs. 19/53  73/94 vs. 17/32  47/95 vs. 15/56 | 51/71 vs. 22/35  63/94 vs. 24/47  33/73 vs. 13/46  21/32 vs. 73/89 |
|  |  | Both fractions have the same gap | 45/64 vs. 75/94  11/72 vs. 37/98  11/36 vs. 22/47  46/61 vs. 17/32 | 26/79 vs. 40/93  72/97 vs. 32/57  35/68 vs. 50/83  74/95 vs. 26/47 | 21/68 vs. 43/90  46/97 vs. 16/67  73/89 vs. 39/55  29/46 vs. 62/79 |
|  | Incongruent | Leads to the correct answer | 39/52 vs. 45/76  23/94 vs. 16/33  43/56 vs. 51/94  53/65 vs. 71/99  28/39 vs. 48/85  62/91 vs. 57/73  23/36 vs. 42/79  45/58 vs. 51/77  43/54 vs. 59/93  31/44 vs. 61/99  39/52 vs. 45/88  35/99 vs. 24/53 | 35/56 vs. 46/85  52/99 vs. 36/47  37/50 vs. 56/97  31/42 vs. 47/93  40/51 vs. 46/83  15/43 vs. 26/97  49/95 vs. 32/43  17/36 vs. 27/73  18/37 vs. 23/74  55/69 vs. 62/97  42/79 vs. 26/37  26/43 vs. 49/94 | 25/88 vs. 18/41  53/98 vs. 37/48  55/98 vs. 31/43  35/46 vs. 41/79  28/41 vs. 55/94  41/57 vs. 46/81  46/91 vs. 30/41  62/99 vs. 45/56  52/91 vs. 47/58  21/77 vs. 15/34  58/85 vs. 39/50  51/98 vs. 34/45 |
|  | Neutral | Leads to the correct answer | 18/49 vs. 12/59  31/78 vs. 17/96  39/77 vs. 46/69  26/79 vs. 19/84  17/59 vs. 25/54  36/77 vs. 31/82  23/99 vs. 37/92  20/43 vs. 14/61  16/79 vs. 22/71  25/54 vs. 17/75  37/65 vs. 43/59  52/97 vs. 67/87 | 49/76 vs. 44/83  59/96 vs. 78/91  35/87 vs. 30/97  13/91 vs. 19/52  55/97 vs. 61/91  23/48 vs. 13/56  31/86 vs. 25/92  61/99 vs. 73/91  13/74 vs. 22/51  48/83 vs. 54/71  26/63 vs. 14/83  23/81 vs. 32/69 | 61/88 vs. 67/83  61/83 vs. 54/97  41/74 vs. 49/63  11/85 vs. 19/78  17/52 vs. 22/45  37/98 vs. 44/89  22/93 vs. 29/71  25/92 vs. 33/86  26/55 vs. 20/91  25/78 vs. 13/93  57/74 vs. 49/94  21/80 vs. 15/94 |

**Table S2. Performance by components, congruency, and experimental block.** Mean accuracies and response times for each of the item types defined by components and congruency, computed separately for the first and last blocks of items of the experimental session. Blocks 1 and 3 comprise items 1-60 and 121-180, respectively. Standard deviations in parentheses.

| **Components** | **Congruency** | **Accuracy** | **Response time [ms]** |
| --- | --- | --- | --- |
| With a common component | Congruent | B1: 98% (6%)  B3: 98% (4%) | B1: 2880 (1029)  B3: 2603 (645) |
|  | Incongruent | B1: 98% (4%)  B3: 97% (5%) | B1: 3149 (998)  B3: 2595 (655) |
| Without common components | Congruent | B1: 84% (16%)  B3: 84% (17%) | B1: 4629 (1595)  B3: 3968 (1178) |
|  | Incongruent | B1: 92% (9%)  B3: 90% (10%) | B1: 4485 (1445)  B3: 3695 (1080) |
|  | Neutral | B1: 97% (5%)  B3: 98% (5%) | B1: 4083 (1193)  B3: 3461 (937) |

# Data and analysis script

The full set of data reported in this study and the analysis script are available in the Open Science Foundation servers at <https://osf.io/nhtkw/>.
